# Non‐Weightbearing in Hinged‐Knee Braces Locked in High Degrees of Flexion Shows High Hamstring Muscle Activation in Healthy Volunteers

**DOI:** 10.1002/ars2.70081

**Published:** 2026-09-09

**Authors:** Christian E. Athanasian, Brendan Schwartz, Natalia Czerwonka, David P. Trofa, Richard C. Mather, Robert A. Christian

**Affiliations:** ^1^ Department of Orthopaedic Sports Medicine Columbia University New York New York U.S.A.; ^2^ Department of Orthopaedic Sports Medicine Duke University Durham North Carolina U.S.A.

## Abstract

**Purpose:**

To determine the effect of various postoperative ambulation and bracing protocols on hamstring muscle group activation in healthy volunteers.

**Methods:**

Hamstring muscle group activation was assessed via Myontec Mbody 3 Shorts. Volunteers ambulated 10 yards in conditions including walking unassisted, flat‐foot weightbearing (FFWB), FFWB with a Newport abduction hip brace restricting motion past 90° of flexion, FFWB with an unlocked hinged‐knee brace, and non‐weightbearing (NWB) in hinged‐knee braces locked in 90°, 60°, and 30° of flexion. Data was standardized with the unassisted walking condition as the reference. Each condition was tested on both extremities. Primary outcomes were normalized cumulative hamstring activation and normalized peak hamstring activation on the protected extremity. Analysis of variance and post hoc Tukey tests were performed.

**Results:**

Fifteen healthy volunteers with no history of hip or knee surgery were recruited over a 1‐year period from June 2023 to June 2024. Analysis of variance testing showed statistically significant differences in both cumulative ipsilateral hamstring muscle activation (*P* = .002) and peak ipsilateral hamstring muscle activation (*P* = .004). Post hoc Tukey testing showed significant differences in cumulative ipsilateral hamstring muscle activation between NWB hinged‐knee braces locked at 90° compared with 30° (3.38 (standard deviation [SD]: 4.03) vs 1.49 (SD: 2.13), *P* = .01) and NWB hinged‐knee braces locked at 90° compared with FFWB with unlocked hinged‐knee braces (3.38 (SD: 4.03) vs 1.54 (SD: 1.15), *P* = .01). For normalized peak muscle activation, significant differences were noted from NWB hinged‐knee braces locked at 60° compared with FFWB (1.12 (SD: 1.21) vs 0.58 (SD: 0.50), *P* = .02), and NWB hinged‐knee braces locked at 60° compared with FFWB with hinged‐knee braces unlocked (1.12 (SD: 1.21) vs 0.61 (SD: 0.57), *P* = .03).

**Conclusions:**

Healthy volunteers showed lower levels of cumulative hamstring muscle activation during FFWB with an unlocked hinged‐knee brace compared with NWB with hinged‐knee braces locked at 90° and NWB with hinged‐knee braces locked at 30° compared with NWB with hinged‐knee braces locked at 90°. Lower levels of peak hamstring muscle activation were noted during FFWB compared with NWB hinged‐knee braces locked at 60° and FFWB with an unlocked hinged‐knee brace compared with NWB hinged‐knee braces locked at 60°.

**Level of Evidence:**

Level IV, retrospective case series.

## Graphical abstract

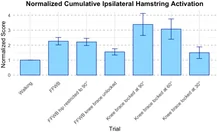

Proximal hamstring avulsions are relatively uncommon injuries, comprising approximately 10% of all hamstring injuries, typically resulting from eccentric contraction during simultaneous hip flexion and knee extension during activities such as sprinting.^1^, ^2^ Proximal hamstring avulsions occur most commonly in middle‐aged patients, with men frequently affected between the ages of 30 and 60 as well as women between the ages of 45 and 60.^3^ Current guidelines favor surgical repair of tears involving all 3 tendons in active patients younger than 60 years of age and 2 tendon tears if retraction is greater than 2 cm.^4^, ^5^ Operative treatment of proximal hamstring avulsions is associated with significantly higher patient satisfaction, hamstring strength, lower extremity functional scale scores, return to preinjury sport level, and single‐legged hop test results when compared with nonoperative treatment.^6^, ^7^ For patients who do undergo proximal hamstring tendon repair, optimal postoperative bracing and weightbearing protocols remain unknown. Postoperative bracing is theorized to be beneficial via decreasing tension on the repair site and providing protection of the surgical repair.

In this study, participants completed conditions including flat‐foot weightbearing (FFWB), FFWB with a Newport hip abduction brace restricting motion past 90° of flexion, FFWB with an unlocked hinged‐knee brace, and non‐weightbearing (NWB) with a hinged knee brace locked at 90° of flexion, 60° of flexion, and 30° of flexion, respectively. Outcome measures included cumulative hamstring muscle activation and peak hamstring muscle activation over a standardized distance of 10 yards, normalized to each individual participant via the completion of a walking condition. The purpose of this study was to determine the effect of various postoperative ambulation and bracing protocols on hamstring muscle group activation in healthy volunteers. We hypothesized that conditions involving FFWB, regardless of use of a brace, would lead to the lowest levels of cumulative hamstring activation and peak hamstring activation.

## METHODS

This study was reviewed and approved by the Institutional Review Board (IRB #: AAAU3653). Fifteen healthy volunteers aged 22 to 61 years were enrolled in this prospective study. No previous pilot studies were conducted that enabled an a priori power analysis. Volunteers were recruited over a 1‐year period from June 2023 to June 2024 at Columbia University Medical Center via advertisements on campus. Exclusion criteria included a history of prior hip or knee injury or surgery and the requirement of an assistive ambulation device for daily ambulation. Height and weight were measured for each patient. Volunteers were fitted for Myontec Mbody 3 Shorts in accordance with their sizing protocols. Measurement of electromyography (EMG) activity with textile electrodes has previously been validated as an alternative to surface EMG.^8^, ^9^ Axillary crutches were adjusted to each volunteer's height. Participants first walked a standardized distance of 10 yards as a control variable. Volunteers then ambulated the same standardized distance with the following weightbearing parameters and brace types: FFWB, FFWB with a Newport abduction hip brace restricting motion past 90° of flexion, FFWB with an unlocked hinged‐knee brace, NWB with a hinged‐knee brace locked in 90° of flexion, NWB with a hinged‐knee brace locked in 60° of flexion, and NWB with a hinged‐knee brace locked in 30° of flexion. Each condition was tested on both right and left extremities. The lead author (C.E.A.), who is well versed in postoperative ambulation protocols, trained participants on the proper technique for FFWB, noting that no more than 20% of the participant's body weight as shown by the use of the weight scale should be placed through the protected extremity and that no hip extension of the protected extremity should be allowed during ambulation. C.E.A. also fitted and instructed participants in the proper use of braces during ambulation conditions. C.E.A. placed the braces on all patients. Hamstring group muscle activation of the protected extremity was measured. Data from each condition were standardized with the walking condition acting as the reference, with participants instructed to walk with their normal gait. This condition was fully weightbearing and a nonbraced condition. The primary outcome measures were normalized cumulative hamstring muscle group activation and normalized peak hamstring muscle group activation of the protected extremity. Cumulative hamstring muscle activation was defined as the total hamstring muscle activation in the protected extremity over the duration of the condition tested needed to complete the standardized distance and was measured in millivolt‐seconds. Program software generated this value as an area under the curve. Peak hamstring muscle activation was defined as the maximal activation recorded over the duration of the condition needed to complete the standardized distance and was measured in millivoltss. Data was normalized by dividing the outcome measure of interest in the specific ambulation condition group by the outcome measure of interest in the initial walking condition that served as the reference condition for that participant, generating a unit‐less number. Descriptive statistics were computed, and 1‐way analysis of variance testing and post hoc Tukey testing were conducted using R, developed by R Foundation (Vienna, Austria). Statistical significance was defined as *P* < .05 for initial analysis of variance testing, and for post hoc Tukey testing, statistical significance was also set at *P* < .05.

## RESULTS

A total of 15 participants and 30 trials (1 per leg) were included. Sample demographics can be seen in Table 1. There were more men (n = 12, 80%) than women (n = 3, 20%) included in the study cohort. The average age of volunteers was 26.1 (range 22‐61); the average body mass index was 23.3 (range 19.9‐28.9); the average height was 68.7 inches (range 62‐74); the average weight was 157.3 pounds (range 120‐198). Three participants wore the small shorts (20%), 5 wore the medium shorts (33%), 6 participants wore the large shorts (40%), and 1 participant wore the extra‐large shorts (6.7%). Normalized cumulative muscle activation is presented as a graph in Figure 1, and numerical values are presented in Table 2. Mean scores were 1.00 for walking, 2.26 (SD: 1.36) for FFWB, 2.21 (SD: 1.31) for FFWB with a hip brace restricting motion past 90° of flexion, 1.54 (SD: 1.15) for FFWB with an unlocked knee brace, 3.38 (SD: 4.03) for NWB with a knee brace locked at 90° of flexion, 3.07 (SD: 3.71) for NWB with a knee brace locked at 60° of flexion, and 1.49 (SD: 2.13) for NWB with a knee brace locked at 30° of flexion. Normalized peak muscle activation is presented as a graph in Figure 2, and numerical values can be seen in Table 2. Mean scores were 1.00 for walking, 0.58 (SD: 0.50) for FFWB, 0.63 (SD: 0.37) for FFWB with a hip brace restricting motion past 90° of flexion, 0.61 (SD: 0.57) for FFWB with an unlocked knee brace, 1.01 (SD: 0.76) for NWB with a knee brace locked at 90° of flexion, 1.12 (SD: 1.21) for NWB with a knee brace locked at 60° of flexion, and 0.73 (SD: 0.78) for NWB with a knee brace locked at 30° of flexion.

**TABLE 1:** Sample Demographics (n = 15 Participants)

	Mean	Median	Range
Age	26.1	23	22‐61
Height, inches	68.7	69	62‐74
Weight, pounds	157.3	154	120‐198
Body mass index	23.3	23.4	19.9‐28.9

	n	%	
Sex			
Male	12	80	
Female	3	20	
Size			
Small	3	20	
Medium	5	33.3	
Large	6	40	
Extra large	1	6.7	

*Note*: Sample demographics of the volunteer population, which consisted of 15 volunteers, by age, height, weight, body mass index, sex, and size of transcutaneous electromyography shorts.

**FIGURE 1:**
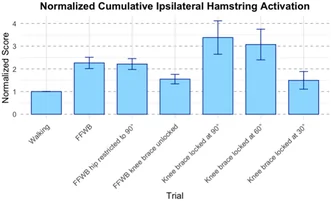
Means are shown with standard error bars. The *x*‐axis shows the protocol used, and the *y*‐axis shows the normalized score for cumulative ipsilateral hamstring activation. Statistically significant differences were noted between NWB hinged‐knee braces locked at 90° compared with 30° (3.38 (SD: 4.03) vs 1.49 (SD: 2.13), *P* = .01) and NWB hinged‐knee brace locked at 90° compared with FFWB with unlocked hinged‐knee brace (3.38 (SD: 4.03) vs 1.54 (SD: 1.15), *P* = .01). (FFWB, flat‐foot weightbearing; NWB, non‐weightbearing; SD, standard deviation.)

**TABLE 2:** Means and Standard Deviations by Condition

Condition Type	Mean (Standard Deviation)
Normalized cumulative ipsilateral hamstring activation	
Walking	1.00 (0)
FFWB	2.26 (1.36)
FFWB with hip brace restricting motion past 90° of flexion	2.21 (1.31)
FFWB with unlocked knee brace	1.54 (1.15)
NWB knee brace locked at 90° of flexion	3.38 (4.03)
NWB knee brace locked at 60° of flexion	3.07 (3.71)
NWB knee brace locked at 30° of flexion	1.49 (2.13)
Normalized peak ipsilateral hamstring activation	
Walking	1.00 (0)
FFWB	0.58 (0.50)
FFWB with hip brace restricting motion past 90° of flexion	0.63 (0.37)
FFWB with unlocked knee brace	0.61 (0.57)
NWB knee brace locked at 90° of flexion	1.01 (0.76)
NWB knee brace locked at 60° of flexion	1.12 (1.21)
NWB knee brace locked at 30° of flexion	0.73 (0.78)

*Note*: Means and standard deviations by condition tested, with primary outcome variables being normalized cumulative ipsilateral hamstring activation and normalized peak ipsilateral hamstring activation.

FFWB, flat‐foot weightbearing; NWB, non‐weightbearing.

**FIGURE 2:**
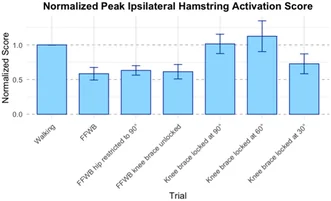
Means are shown with standard error bars. The *x*‐axis shows the protocol used, and the *y*‐axis shows the normalized score for peak ipsilateral hamstring activation. Statistically significant differences were noted from NWB hinged‐knee brace locked at 60° compared with FFWB (1.12 (SD: 1.21) vs 0.58 (SD: 0.50), *P* = .02) and NWB hinged‐knee brace locked at 60° compared with FFWB with hinged‐knee brace unlocked (1.12 (SD: 1.21) vs 0.61 (SD: 0.57), *P* = .03). (FFWB, flat‐foot weightbearing; NWB, non‐weightbearing; SD, standard deviation.)

Analysis of variance testing showed statistically significant differences in both cumulative ipsilateral hamstring muscle activation (*P* = .002) and peak ipsilateral hamstring muscle activation (*P* = .004). Post hoc Tukey testing for pairwise comparisons showed statistically significant differences in cumulative ipsilateral hamstring muscle activation between the following bracing protocols: NWB with a hinged‐knee brace locked at 90° compared with NWB with a hinged knee brace locked at 30° (3.38 (SD: 4.03) vs 1.49 (SD: 2.13), *P* = .01) and NWB with a hinged‐knee brace locked at 90° compared with FFWB with unlocked hinged‐knee brace (3.38 (SD: 4.03) vs 1.54 (SD: 1.15), *P* = .01). Statistically significant differences in peak ipsilateral muscle activation were noted between the following bracing and weightbearing conditions: NWB with a hinged‐knee brace locked at 60° compared with FFWB (1.12 (SD: 1.21) vs 0.58 (SD: 0.50), *P* = .02) and NWB with a hinged‐knee brace at 60° compared with FFWB with hinged‐knee brace unlocked (1.12 (SD: 1.21) vs 0.61 (SD: 0.57), *P* = .03). These can be seen in Table 3.

**TABLE 3:** Post Hoc Tukey *P* Values

Pairwise Comparisons	Ipsilateral Cumulative Hamstring Activation	Ipsilateral Peak Hamstring Activation
FFWB vs FFWB with hip brace restricting motion past 90° of flexion	>.99	>.99
FFWB vs FFWB with unlocked knee Brace	.77	>.99
FFWB vs NWB knee brace locked at 90° of flexion	.31	.11
FFWB vs NWB knee brace locked at 60° of flexion	.66	.02*
FFWB vs NWB knee brace locked at 30° of flexion	.71	.96
FFWB with hip brace restricting motion past 90° of flexion vs FFWB with unlocked knee brace	.82	>.99
FFWB with hip brace restricting motion past 90° of flexion vs NWB knee brace locked at 90° of flexion	.26	.21
FFWB with hip brace restricting motion past 90° of flexion vs NWB knee brace locked at 60° of flexion	.60	.05
FFWB with hip brace restricting motion past 90° of flexion vs NWB knee brace locked at 30° of flexion	.77	.99
FFWB with unlocked knee brace vs NWB knee Brace locked at 90° of flexion	.01*	.17
FFWB with unlocked knee brace vs NWB knee brace locked at 60° of flexion	.06	.03*
FFWB with unlocked knee brace vs NWB knee brace locked at 30° of flexion	>.99	.98
NWB knee brace locked at 90° of flexion vs NWB knee brace locked at 30° of flexion	.01*	.52
NWB knee brace locked at 60° of flexion vs NWB knee brace locked at 30° of flexion	.05	.17

*Note*: Post hoc pairwise comparison across the different conditions tested, with primary outcome variables being normalized ipsilateral cumulative hamstring activation, and normalized ipsilateral peak hamstring activation. Statistically significant results are bolded and denoted with an asterisk.

FFWB, flat‐foot weightbearing; NWB, non‐weightbearing.

## DISCUSSION

In this study, healthy volunteers showed lower levels of cumulative hamstring muscle activation during FFWB with an unlocked hinged‐knee brace compared with NWB with hinged‐knee braces locked at 90° and NWB with hinged‐knee braces locked at 30° compared with NWB with hinged‐knee braces locked at 90°. Lower levels of peak hamstring muscle activation were noted during FFWB compared with NWB with hinged‐knee braces locked at 60° and FFWB with an unlocked hinged‐knee brace compared with NWB with hinged‐knee braces locked at 60°.

Proximal hamstring avulsions are debilitating to patients and athletes, resulting in significant time off work and long periods of inability to participate in sports.^5^ A 2018 meta‐analysis reported improved lower extremity functional scale scores, hamstring strength, and patient satisfaction with surgical repair compared with nonoperative treatment.^6^ However, there is no consensus for postoperative bracing protocols for patients undergoing proximal hamstring tendon repair.

In a cross‐sectional study exploring online proximal hamstring physical therapy protocols from academic US hospitals, Lightsey et al. identified 35 complete proximal hamstring repair rehabilitation protocols, of which 71% recommended immediate postoperative bracing, 34% recommended knee bracing, 23% recommended hip bracing, and 14% did not specify the type of brace recommended. Of those recommending knee bracing, there was variability in the angle of the knee brace locked in flexion ranging from 45° to 90°, with 90° being the most common (64%).^10^ Knee braces can also be locked in lower degrees of flexion and even locked in full extension as shown by Wong et al.^11^ Weightbearing protocols also vary between NWB, FFWB, and weight‐bearing as tolerated.

The need for any form of postoperative bracing after proximal hamstring repair was questioned in a 2019 case series of 57 patients who were not braced after proximal hamstring tendon repair, showing low rates of retear.^12^ A 2025 randomized controled trial of 57 patients comparing patients who were braced with a knee brace worn at all times fixed at 30° with toe‐touch weightbearing for the first 2 weeks followed by partial weightbearing for the next 4 weeks to an accelerated rehabilitation group that allowed full weightbearing as tolerated with the initial support of elbow crutches found no differences in patient satisfaction, knee flexor torque, reinjuries, or complications at 6 months. Two reruptures occurred, both of which were in the conservative group.^13^ Conversely, a 2024 systematic review comparing 905 patients who were braced postoperatively and 291 patients who were not braced postoperatively after proximal hamstring tendon repair found slightly lower complication rates and reoperation rates, higher patient‐reported outcomes scores, higher patient satisfaction, and a higher return to sport rate in braced patients compared with nonbraced patients.^14^ However, bracing and ambulation protocols were not uniform within the compared groups, limiting generalizability of the study. Therefore, it still remains unclear which ambulation and bracing strategy postoperatively is optimal.

The decision for surgeons regarding postoperative bracing and ambulation strategies after proximal hamstring tendon repair includes many factors such as anticipated tension on the repair site, modality used for fixation, friability of the tissue and medical comorbidities and behaviors such as smoking that may influence tissue healing. The results of our study may inform surgeon decision‐making with regard to postoperative ambulation and bracing protocols after proximal hamstring tendon repair. However, it should be noted that although the results of our study could be used to infer hamstring activation during postoperative ambulation, it does not explore tension that may passively be placed on the repair site by passive stretching of the tissues during hip flexion and knee extension. Bracing serves to protect the repair site not only during patient activity but also during passive states such as when a patient lies on a couch.

Based on our studies’ results, instructing the patient to ambulate with FFWB, with or without a brace, may be superior when compared with NWB in a hinged‐knee brace locked in a higher degree of flexion. Whether to brace patients who are FFWB is surgeon preference. When patients place or remove a brace on themselves, they may frequently assume uncomfortable positions such as a “hurdler position” that may put strain on the repair site. Biomechanical studies have shown that repeated events of flexing the hip, especially at 90°, can increase displacement across the proximal hamstring repair site.^15^ Conversely, without a brace, patients may be more likely to enter these positions involving a high degree of hip flexion, such as sitting on a couch with the hip in a high degree of flexion. Braces do not only serve a function during patient activity but also serve to protect the patient during passive states. Overall, it appears reasonable to forgo postoperative bracing after proximal hamstring tendon repair. However, in these situations, surgeons must anticipate common everyday positions patients may passively enter that could put tension on the repair site and counsel them on avoiding positions that may tension the repair site. If a patient is to be braced, it should be recommended that the patient receives assistance from another person when placing and removing the brace.

### Limitations

There are some limitations to our study. First, our study relies on the assumption that hamstring muscle activation, both cumulative and peak, is a proxy for stress and strain at the repair site during the healing phase after proximal hamstring repair. Our study population also consisted of healthy volunteers, as opposed to postoperative proximal hamstring tendon repair patients, and therefore may not accurately reflect the cumulative muscle load and peak muscle activation that these patients experience during postoperative ambulation. We were unable to desegregate the data by sex, as recommended by the sex and gender equity in research guidelines because of the small sample size and the disproportionate number of men in our sample. We believe part of the reason for this pertained to the use of tight‐fitting compression shorts to gain the transcutaneous EMG data, which may have influenced the generalized willingness to participate in the study.

Study results may have also been influenced by participant repetition of different ambulation techniques throughout the study. This may apply specifically to FFWB conditions, as conditions were measured consecutively with FFWB without the use of a brace first, and subsequently those with a hip brace restricted to 90° of flexion and an unlocked knee brace. Additionally, participants may have experienced muscle fatigue because of consecutive trials. Of particular importance to EMG studies is the choice of a reference condition for normalization of data for comparison across different individuals. Therefore, we were reliant on our walking condition as our reference group, and abnormalities from that condition may have influenced our outcome variables as a normalized score. Additionally, our study uses transcutaneous EMG, which is less invasive than traditional EMG but may not be as accurate as traditional EMG data. Lastly, although statistically significant results were observed, no a priori power analysis was conducted, limiting confidence in the precision and replicability of our results.

## CONCLUSIONS

Healthy volunteers showed lower levels of cumulative hamstring muscle activation during FFWB with an unlocked hinged‐knee brace compared with NWB with hinged‐knee braces locked at 90° and NWB with hinged‐knee braces locked at 30° compared with NWB with hinged‐knee braces locked at 90°. Lower levels of peak hamstring muscle activation were noted during FFWB compared with NWB hinged‐knee braces locked at 60° and FFWB with an unlocked hinged‐knee brace compared with NWB hinged‐knee braces locked at 60°.

## DISCLOSURES

The author (R.C.M.) declares the following financial interests/personal relationships which may be considered as potential competing interests: R.C.M. reports that equipment, drugs, or supplies were provided by Duke University Health System and reports a relationship with Stryker that includes consulting or advisory and travel reimbursement and with Surgical Care Affiliates LLC that includes employment. The other authors (C.E.A., B.S., N.C., D.P.T., R.A.C.) declare that they have no known competing financial interests or personal relationships that could have appeared to influence the work reported in this article.

## References

[1] Koulouris G , Connell D . Evaluation of the hamstring muscle complex following acute injury. Skeletal Radiol. 2003;32:582‐589. 10.1007/s00256-003-0674-5 12942206

[2] Looney AM , Day HK , Comfort SM , Donaldson ST , Cohen SB . Proximal hamstring ruptures: Treatment, rehabilitation, and return to play. Curr Rev Musculoskelet Med. 2023;16:103‐113. 10.1007/s12178-023-09821-7 PMC9943812 36757628

[3] Irger M , Willinger L , Lacheta L , Pogorzelski J , Imhoff AB , Feucht MJ . Proximal hamstring tendon avulsion injuries occur predominately in middle‐aged patients with distinct gender differences: Epidemiologic analysis of 263 surgically treated cases. Knee Surg Sports Traumatol Arthrosc. 2020;28:1221‐1229. 10.1007/s00167-019-05717-7 31541291

[4] Fletcher AN , Cheah JW , Nho SJ , Mather RC . Proximal hamstring injuries. Clin Sports Med. 2021;40:339‐361. 10.1016/j.csm.2021.01.003 33673891

[5] Bertiche P , Mohtadi N , Chan D , Hölmich P . Proximal hamstring tendon avulsion: State of the art. J ISAKOS. 2021;6:237‐246. 10.1136/jisakos-2019-000420 34272300

[6] Bodendorfer BM , Curley AJ , Kotler JA , et al. Outcomes after operative and nonoperative treatment of proximal hamstring avulsions: A systematic review and meta‐analysis. Am J Sports Med. 2018;46:2798‐2808. 10.1177/0363546517732526 29016194

[7] Harris JD , Griesser MJ , Best TM , Ellis TJ . Treatment of proximal hamstring ruptures ‐ A systematic review. Int J Sports Med. 2011;32:490‐495. 10.1055/s-0031-1273753 21563032

[8] Finni T , Hu M , Kettunen P , Vilavuo T , Cheng S . Measurement of EMG activity with textile electrodes embedded into clothing. Physiol Meas. 2007;28:1405‐1419. 10.1088/0967-3334/28/11/007 17978424

[9] Colyer SL , McGuigan PM . Textile electrodes embedded in clothing: A practical alternative to traditional surface electromyography when assessing muscle excitation during functional movements. J Sports Sci Med. 2018;17:101‐109. PMC5844196 29535583

[10] Lightsey HM , Kantrowitz DE , Swindell HW , Trofa DP , Ahmad CS , Lynch TS . Variability of United States online rehabilitation protocols for proximal hamstring tendon repair. Orthop J Sports Med. 2018;6:2325967118755116. 10.1177/2325967118755116 PMC5826004 29511700

[11] Wong SE , Julian KR , Carpio JG , Zhang AL . Proximal hamstring repair with all‐suture anchors and an accelerated rehabilitation and bracing protocol demonstrates good outcomes at 1‐year follow‐up. Arthrosc Sports Med Rehabil. 2024;6:100891. 10.1016/j.asmr.2024.100891 PMC10867423 38362482

[12] Lazaro LE , Banffy MB . Is brace immobilization needed following surgical repair of proximal hamstring tears? Orthop J Sports Med. 2019;7:2325967119S00418.

[13] Aujla RS , Edwards P , Cecchi S , et al. Accelerated rehabilitation after proximal hamstring avulsion repair is safe and effective: Outcomes from randomized controlled trial of two different rehabilitation regimes. Knee Surg Sports Traumatol Arthrosc. 2025;33:4412‐4425. 10.1002/ksa.70030 PMC12684348 40923452

[14] Wyatt PB , Ho TD , Hopper HM , et al. Systematic review of bracing after proximal hamstring repair. Orthop J Sports Med. 2024;12:23259671241230045. 10.1177/23259671241230045 PMC10894551 38405008

[15] Harvey MA , Singh H , Obopilwe E , Charette R , Miller S . Proximal hamstring repair strength: A biomechanical analysis at 3 hip flexion angles. Orthop J Sports Med. 2015;3:2325967115576910. 10.1177/2325967115576910 PMC4622336 26665049

